# Innovative Characterization of Particulate Matter Deposited on Urban Vegetation Leaves through the Application of a Chemical Fractionation Procedure

**DOI:** 10.3390/ijerph17165717

**Published:** 2020-08-07

**Authors:** Martina Ristorini, Chiara Baldacchini, Lorenzo Massimi, Gregorio Sgrigna, Carlo Calfapietra

**Affiliations:** 1 Department of Bioscience and Territory, Università degli Studi del Molise, 86090 Pesche (IS), Italy; m.ristorini@studenti.unimol.it; 2 Institute of Research on Terrestrial Ecosystems (IRET), National Research Council (CNR), 05010 Porano (TR), Italy; gregorio.sgrigna@iret.cnr.it (G.S.); carlo.calfapietra@cnr.it (C.C.); 3 Biophysics and Nanoscience Centre, Department of Ecological and Biological Sciences (DEB), Università degli Studi della Tuscia, 01100 Viterbo, Italy; 4 Department of Chemistry, Università degli Studi di Roma “La Sapienza”, Piazzale Aldo Moro, 5, 00185 Roma, Italy; l.massimi@uniroma1.it

**Keywords:** air quality, particulate matter, chemical fractionation, pollution source tracers, nature-based solutions

## Abstract

In this study, we have evaluated the efficiency of a chemical fractionation procedure for the characterization of both the water-soluble and the insoluble fraction of the main elemental components of particulate matter (PM) deposited on urban leaves. The proposed analytical approach is based on the chemical analysis of leaf washing solutions and membrane filters used for their filtration. The ionic concentration of leaf washing solutions was compared with their electrical conductivity, making it a valuable proxy for the quantification of the water-soluble and ionic fraction of leaf deposited PM. The chemical composition of both the water-soluble and the insoluble fraction of PM, resulting from this fractionation procedure, was compared with results obtained by scanning electron microscopy coupled with energy-dispersed X-Rays spectroscopy (SEM/EDX) and processed through chemometrics. Results obtained proved that the proposed approach is able to provide an estimation of total leaf deposited PM and it is highly reliable for the evaluation of the emission impact of different PM sources, being able to increase the selectivity of PM elemental components as specific source tracers; consequently providing useful information also for the assessment of human health risks.

## 1. Introduction

Among the ecosystem services provided by urban vegetation, its potential to reduce air pollution has become one of the most important objects of investigation, being largely discussed in literature [1,2,3]. In urban and industrial areas, particulate matter (PM) pollution is considered one of the biggest concerns regarding human health and well-being, its exposure being correlated mainly to cardiovascular and respiratory disorders [4,5,6]. Airborne PM is a complex mixture of solid particles and liquid droplets characterized by different size, chemical composition, morphology and solubility [7]. Its emission is related both to natural and anthropogenic emission sources (such as vehicular traffic, industrial plants, domestic heating and biomass burning) [8] and its effects on human health are strongly dependent on its size distribution, chemical composition and solubility [9,10,11].

Vegetation mitigates PM pollution, since particles can be trapped on leave surfaces through deposition mechanisms [1,12,13]. However, deposition of airborne particles on leaves is a complex and dynamic process, being influenced by numerous parameters. Leaf deposition efficiency depends on particles characteristics, such as their morphology and size [14], but also on species-specific leaf characteristics (such as leaf area, leaf morphology, density of stomata pores and thickness of wax layers) [15,16,17,18]. In addition, the density and the porosity of the tree crown, as well as meteorological conditions such as precipitations, wind speed and direction, result to be relevant in influencing the process [12,19,20].

The interaction between vegetation and airborne particles has been the object of a growing number of studies in the last years, which focused, among the rest, on the achievement of information on the chemical composition, solubility, size and morphology of leaf deposited particles. Indeed, these chemical and physical characteristics deserve particular interest due to their potential for the identification of emission sources and for the assessment of the risk exposure for human health [21,22,23]. The elemental composition and size distributions of leaf deposited airborne particles have been proved to be highly related with pollution source apportionment [24,25,26,27,28], and the use of leaves of urban trees as air passive filters for PM monitoring is emerging as a valid alternative to traditional air quality monitoring networks [29,30,31]. Indeed, urban trees are widespread, thus providing a high density of potential samplers, corresponding to a higher spatial resolution in PM monitoring with respect to standard air quality monitoring stations [32,33]; this aspect being important for the evaluation of PM emission sources and related human health risks. Moreover, the use of already present trees in the urban context as passive PM samplers results in a substantial reduction of the monitoring activity costs, nullifying those related to the purchase and maintenance of PM sampling instruments. However, to date it is still unclear which is the most efficient analytical procedure applicable to the chemical and physical characterization of leaf deposited particles. Standard spectroscopic techniques such as X-ray fluorescence spectroscopy (XRF), as well as magnetic biomonitoring techniques, such as saturation isothermal remanent magnetization (SIRM), have been largely applied for the detection of leaves elemental concentrations [34,35]. However, these techniques are based on the characterization of the whole leaves, thus making impossible to discriminate whether the detected elements are due to leaf deposited particles or to plant uptake from soil. A different approach has been proposed by Dzierżanowski et al. in 2011 [36]: vacuum filtration (V/F) gravimetric technique is based on leaf washing and subsequent filtration of the washing solution through membrane filters with a decreasing porosity. The weighting of the filters before and after the washing solution filtration allows to gravimetrically assess the amount of leaf deposited PM per unit leaf area into different size fractions, depending on the filter porosity. However, gravimetric V/F has two main limitations: it is not able to provide information on the PM chemical composition (which is essential for source apportionment) and it cannot take into account the soluble part of leaf deposited PM (which is likely dissolved into the washing solution). To date, scanning electron microscopy (SEM) analysis, implemented with energy-dispersed X-ray spectroscopy (EDX), results to be the analytical procedure able to provide the most complete characterization of leaf deposited particles. This technique provides information about the number, size and morphology of PM particles, in addition to information on their elemental composition [27,31,37,38,39]. However, SEM/EDX is affected by a main limitation: long analysis time is required to obtain a statistically valid estimation of the PM characteristics per single sample, due to the spatial scale of the single data collected (hundreds of micron square of leaf area per image).

In this study, a new approach to characterize inorganic leaf deposited PM particles with a diameter smaller than 10 μm (PM_10_), which are able to penetrate the human respiratory system, is presented. It is based on the combination of the V/F technique with a chemical fractionation procedure. The V/F of the leaf washing solutions ensures that only material from the leaf surfaces is analysed. The chemical fractionation procedure, which is known to be effective in increasing the selectivity of elements as tracers of PM emission sources [40,41], will extract the PM elemental concentrations from both the insoluble and the soluble part of the washed material, being applied to both filters and washing solutions, respectively. The procedure has been tested on leaves collected in two urban areas of Italy (Naples and Terni), characterized by the presence of different and high-impact anthropogenic PM emission sources. In Naples, holm oak (*Quercus ilex* L.) leaves were collected from seven sampling sites with different exposure to local PM emission sources, to test the chemical fractionation sensitivity to PM sources. In Terni, leaves were sampled from twelve different tree species and four different shrubs species, in the same urban park and with the same exposition toward a well-known air pollution source, to test the suitability of the different tree species to be used as PM passive filters for monitoring purposes. Firstly, the ionic concentrations of leaf washing solutions have been compared to their electrical conductivity (EC), which is expected to be proportional to the total dissolved solid (TDS) in aqueous solutions [42,43]. Then, results obtained through chemical fractionation have been compared with those from gravimetric V/F and SEM/EDX analyses. Finally, the efficiency of the chemical fractionation procedure in increasing the selectivity of the elemental components of leaf deposited PM as specific source tracers was tested through the application of principal component analysis (PCA) applied to the water-soluble and insoluble elemental concentrations. The results obtained in this study prove the reliability of chemical fractionation in the characterization of inorganic leaf deposited PM, as obtained by the V/F procedure, and they represent a further validation for the utilization of leaves as low-cost PM passive filters. Nevertheless, an important warning arises from our results with respect to the choice of the tree species: unreliable data could be obtained from some tree species, likely due to the release of leaf biological material in the washing solutions.

## 2. Materials and Methods

### 2.1. Study Areas

Leaves were collected from two urban hot-spots in Italy, characterized by the presence of several PM emission sources, either natural or anthropogenic. The first set of leaves was collected from the urban forest “Real Bosco di Capodimonte” in Naples (Southern Italy). Naples is a densely populated city characterized by typical urban emission sources such as heavy load of vehicular traffic and domestic heating but also by major industrial plants within the urban area [44]. A second set of leaves was collected in Terni, an urban and industrial hot-spot of Central Italy [27,45,46,47]. In addition to typical PM urban emission sources, such as vehicular traffic and domestic heating, Terni is characterized by the presence of a wide steel plant in the East of the city and an incinerator in the West [48]. The important presence of anthropogenic PM emission sources, in combination with the peculiar meteorological conditions of the city, located within an intramountain valley, determines the ideal conditions for increasing the accumulation of airborne particles and reducing their dispersion [49].

### 2.2. Leaf Sampling

**Figure 1:**
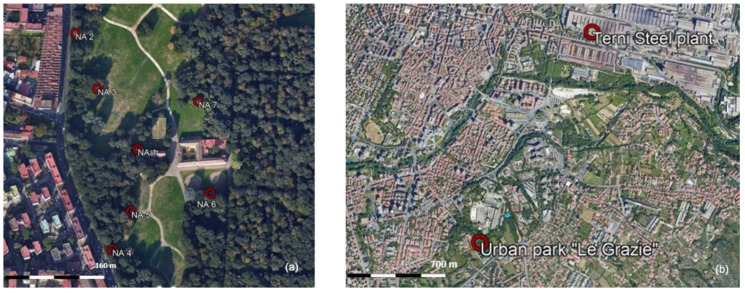
(**a**) Location of the seven sampling sites within the urban forest *“Real Bosco di Capodimonte”* in Naples (Italy). (**b**) Location of the urban park *“Le Grazie”* in Terni (Italy), with respect to the main air pollution source (i.e., the steel plant).

In Naples, leaves of holm oak (*Quercus ilex* (L.)), were collected on February, 1st 2017, from seven different sampling sites (NA 1, NA 2, NA 3, NA 4, NA 5, NA 6, NA 7) within the “Real Bosco di Capodimonte” (see Figure 1a): NA 1 within the wood, NA 2 and NA 4 nearest to a high traffic street, NA 3 and NA 5 located between the wood and the meadow, and NA 6 and NA 7 between the wood and a brownfield. A more detailed description of the Naples sampling sites is reported in Baldacchini et al. 2019 [31]. In Terni, leaves were collected from twelve tree species (*Acer saccharinum* L., *Catalpa bignonioides* Walter, *Cedrus atlantica* (Endl.) Manetti ex Carrière, *Celtis australis* L., *Magnolia grandiflora* L., *Platanus acerifolia* (Aiton) Willd., *Populus nigra* L., *Populus tremula* L., *Prunus cerasifera* Ehrh., *Quercus pubescens* Willd., *Robinia pseudoacacia* L. and *Tilia cordata* Mill.), three shrubs species (*Laurus nobilis* L., *Laurus cerasus* L., *Nerium oleander* L.) and *Hedera helix* L., at high proximity from each other within the urban park “Le Grazie” (42°33′3.18″ N, 12°39′2.03″ E) on June, 15th 2017. A more detailed description of the sampling site and sampled species is reported in Sgrigna et al. 2020 [18]. The leaves have been collected, both in Naples and Terni, more than 15 days after the last intense (i.e., precipitation rate higher than 10 mm h^−1^) rain event.

Two replicate branches were collected per each sampled plant, both in Naples and in Terni. Sampling height was set at 6 m for trees and at 2 m for shrubs. In Terni, for *H. Helix* (L.), two replicate branches were collected at two different heights (these are reported as *H.e. A* and *H.e. B*), comparable both to tree and shrub samples. In order to ensure homogeneity among the leaves sampled and avoid potential influence of their age (which could affect the time exposure to airborne PM pollution and the amount of particles deposited [50]), only the youngest ones were selected in both locations, thus sampling the leaves at the top of the branches [18,31].

All the replicate branches were collected from the external part of the crown, at specific cardinal directions. In Naples, the cardinal direction was selected either toward the sea breeze from South-West (sites NA 1, NA 4, NA 5, NA 7), or toward the land wind from North-West (sites NA 2, NA 3, NA 6) [31]. In Terni, all the species were sampled from the East-North-East part of the crown, towards the wind direction coming from the steel plant (see Figure 1b) [27], in order to select leaves under similar conditions, e.g., influenced by the same pollution source [18]. Leaves collected in both locations were preserved in paper bags to avoid external contamination and stored in freezer (−20 °C).

### 2.3. Vacuum Filtration

For the application of V/F procedure, between 300 and 700 cm^2^ of total leaf area were selected from sampled branches; the number of leaves varied depending on the leaf dimensions. Leaves were washed in micro-filtered (0.2 µm) and deionized water (250 mL and 500 mL for Naples and Terni samples, respectively) inside a flask, and hand-shacked for 5 min. Since we are interested in study PM_10_ particles, washing solutions were first filtrated through a 100 µm porosity sieve, to remove the coarsest material. Through a vacuum filtration system, leaf washing solutions were consequently filtrated with cellulose filters able to remove particles with diameter larger than 10 µm (code 1250; smallest stopped particle size in the 10–13 µm range, Anoia S.A., Barcelona, Spain) and larger than 2 µm (code 1244; smallest stopped particle size in the 2–4 µm range, Anoia S.A.). As a consequence, particles with diameter between about 2.5 µm and 10 µm (i.e., PM_2.5-10_) can be assumed to accumulate on this second filter. Then, washing solutions were filtered with a 0.2 µm porosity nitrocellulose filter (type CN, LVR > 7, Advanced Microdevices Pvt. Ltd., Ambala Cantt, India), where particles with a diameter ranging from 0.2 µm to about 2.5 µm (i.e., PM_0.2–2.5_) are expected to accumulate. After filtration, the washing solutions were collected in falcon tubes and then processed with the chemical fractionation procedure.

To obtain the gravimetric determination of PM loads, membrane filters were weighted before and after the filtration (R200D Research Analytical Balance, Sartorius, Gottingen, Germany), and the difference between the two values was assumed to be the mass of the accumulated PM. To equilibrate humidity levels, membrane filters were dried at 65 °C for 40 min in a moisture control oven (Griffin Company, Paoli, PA, USA) before and after the sequential filtration of washing solutions and kept for 30 min into the balance room before weighting. PM mass for each size class were then normalized by the cm^2^ of total two-sided area of washed leaves (both abaxial and adaxial surfaces), measured through the optical scanning of the washed leaves and then using ImageJ open source software [51]. This procedure allowed to obtain a quantification (in terms of mg cm^−2^) of insoluble PM load per unit leaf area of PM_2.5–10_ and PM_0.2–2.5_ that, summed together, provide the total PM_10_ load per unit leaf area, as reported in Baldacchini et al. [31] and Sgrigna et al. [18]. The same filters used for the gravimetric determination of PM_2.5–10_ and PM_0.2–2.5_ loads were then processed with the chemical fractionation procedure.

### 2.4. Chemical Characterization of Washing Solutions

Leaf washing solutions were analysed by ionic chromatography (IC, ICS1000; Dionex Co., Sunnyvale, CA, USA) for the detection of Cl^−^, F^−^, NO_3_^2−^, PO_4_^3−^ and SO_4_^2−^ and by inductively coupled plasma mass spectrometry (ICP-MS, Bruker 820, Bremen, Germany) for the detection of the concentration of 35 elements (Al, As, B, Ba, Ca, Cd, Ce, Co, Cr, Cs, Cu, Fe, Ga, K, La, Li, Mg, Mn, Mo, Na, Nb, Ni, P, Pb, Rb, Sb, Si, Sn, Sr, Tl, Ti, U, V, W, Zn). The analytical and instrumental conditions used for IC and ICP-MS analysis were previously reported by Canepari et al. [40,41] and by Astolfi et. al. [52], repectively. In addition, ammonium (NH_4_^+^) was detected by UV-Visible spectrophotometer (50 Scan Varian, Santa Clara, CA United States ), following the procedure indicated in the Ammonium-Test, Spectroquant (Merck, Darmstadt, Germany). The chemical characterization of leaf washing solutions was completed by the determination of the water-soluble organic carbon (WSOC), analysed by TOC-VSCH (Shimadzu, Kyoto, Japan) using the non-purgeable organic carbon (NPOC) procedure reported in Saarikoski et al. [50]. Firstly, the concentrations of anionic species such as Cl^−^, F^−^, NO_3_^2−^, PO_4_^3−^ and SO_4_^2−^, obtained through IC, and cationic species such as Na^+^, Ca^2+^, K^+^, Mg^2+^ and NH_4_^+^, obtained through ICP-MS and UV-Vis spectrophotometer, were used to evaluate the ionic balance of each washing solution. Specifically, the ionic balance was evaluated comparing the μmoles of cations (sum of Na^+^, Ca^2+^, K^+^, NH_4_^+^ and Mg^2+^ μmoles) and the μmoles of anions (sum of Cl^−^, F^−^, NO_3_^2−^, PO_4_^3−^, CO_3_^2−^ and SO_4_^2−^ μmoles) for each washing solution, then averaged over the two replicates for each sampling site in Naples and each Terni species. Since CO_3_^2−^ could not be detected through our analytical procedure, its concentration was estimated assuming the balance between μmoles of Ca^2+^ and μmoles of CO_3_^2−^.

Total ionic concentration (∑ Ions in mg L^−1^) was compared to the EC of leaf washing solutions, as obtained by a conductimeter equipped with standard 5070 platinum cell (Crison Basic 30, Crison Instruments, Barcelona, Spain), to verify if a linear relationship exists between them and to quantify the conversion coefficient. Indeed, *EC* of aqueous solutions is known to be proportional to the concentration of *TDS*, according to Equation (1), with the conversion coefficient *K* being dependent on the ion valency [43,53,54]:

(1) $$TDS\;\left(mg\;L^{-1}\right)=constant+(K\;\times \;EC\;\left(\mu S\;cm^{-1}\right)$$

All the concentrations detected in the washing solutions (ions and elements expressed in mg L^−1^) were further multiplied by the volume of the leaf washing solutions, then normalized by the total two-sided washed leaf area measured for each replicate, and averaged for each Naples sampling site and each species sampled in Terni, in order to obtain the concentration of water-soluble species per unit leaf area (mg cm^−2^).

### 2.5. Chemical Characterization of Membrane Filters

For each replicate branch, the filters with accumulated PM_2.5–10_ and PM_0.2–2.5_ were transferred into polytetrafluoroethylene (PTFE) vessels, mixed with 2 mL HNO_3_ (67%; Promochem, LGC Standards GmbH, Wesel, Germany), 1 mL HF (40%, Suprapur, Merck) and 3 mL HCl (puriss. p.a., Sigma-Aldrich, Co., St. Louis, MO, USA) and subsequently heated in microwave oven (Ethos Touch Control, Milestone, Sorisole, Italy) to 180°C for 40 min, as in previously reported method [55]. The acid-digested solutions were then diluted in 10 mL of deionized water. The concentrations of 15 elemental components (Al, Ba, Ca, Cr, Cu, Fe, K, Mg, Mn, Mo, Na, P, Si, Sr, Ti) were determined with inductively coupled plasma atomic emission spectroscopy (ICP-OES; VISTA-MPX, CCD Simultaneous, Varian) equipped with an inert introduction line. The instrumental conditions for ICP-OES analysis were described in a previous study in detail [55]. Elemental concentrations were normalized by the total two-sided washed leaf area measured per each replicate and then averaged over each Naples sampling site and each tree species sampled in Terni (shrubs and *Hedera helix* have not been processed at this stage). This procedure allowed us to obtain the elemental concentrations of the insoluble fraction of inorganic leaf deposited particles per leaf area unit (mg cm^−2^) in two size classes: fine (PM_0.2–2.5_) and coarse (PM_2.5–10_) particles. Then, elemental concentrations per unit leaf area detected in both filters were summed to obtain the load of the inorganic insoluble fraction of leaf deposited PM (mg cm^−2^) per each size class (PM_2.5_, PM_2.5–10_).

### 2.6. Scanning Electron Microscopy Analysis

Two leaves from each replicate branch of Naples samples were analysed by a scanning electron microscope (Phenom ProX, Phenom-World, Eindhoven, Netherlands) coupled with an X-ray analyser and equipped with a charge-reduction sample holder suited for biological samples. By combining SEM images of leaf surfaces and EDX spectra of leaf deposited particles, the elemental concentrations per unit leaf area in the PM particles observed and the total leaf PM load (both in mg cm^−2^), per size fraction, have been obtained, as described in Baldacchini et al., in which results relative to SEM/EDX microanalysis of Naples samples were also reported [31].

### 2.7. Statistical Analysis

Pearson’s coefficients were calculated to highlight statistically significant correlations between the results achieved through the different analytical procedures (chemical fractionation, V/F, SEM/EDX). PCAs were performed on Naples and Terni datasets, using as input variables water-soluble and insoluble elemental concentrations. Both Pearson’s coefficients and PCA were obtained using Statistica v 8 (StatSoft Italia srl, Padua, Italy). Graphical outputs were produced by using OriginPro 8.6 (OriginLab, Northampton, MA, USA).

## 3. Results and Discussion

### 3.1. PM soluble Fraction: Chemical Characterization of Leaf Washing Solutions and Relationship with Electrical Conductivity

**Figure 2:**
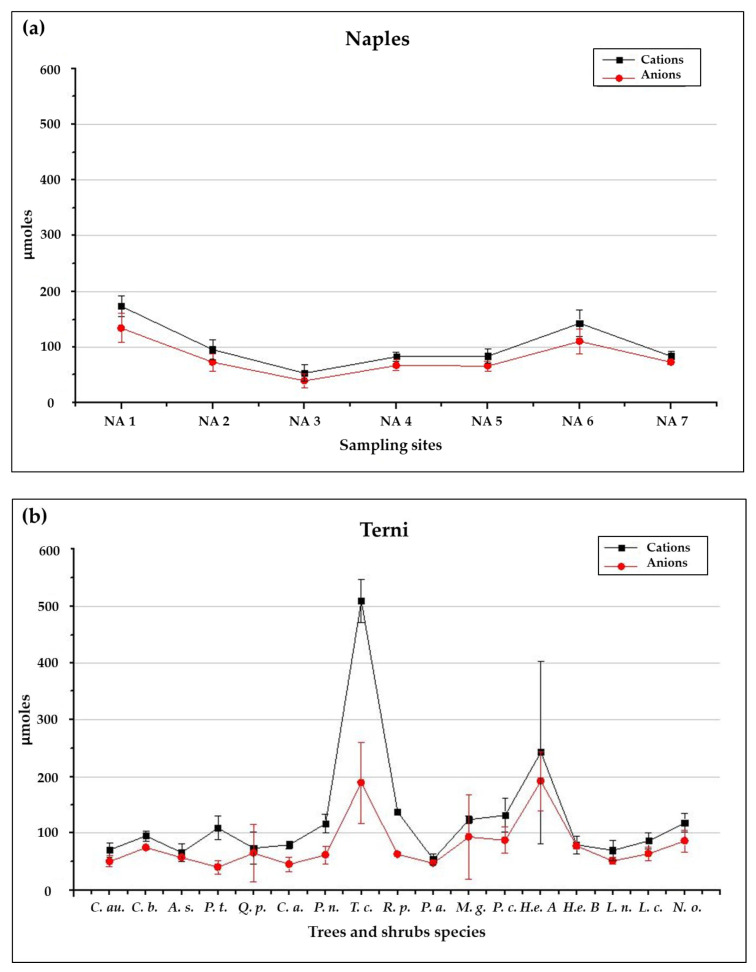
(**a**) Ionic balance of leaf washing solutions averaged over the two replicate branches of each sampling site in Naples; (**b**) Ionic balance of leaf washing solutions averaged over the two replicate branches of each tree and shrub species from Terni. Standard deviation between the two replicates are reported.

A good ionic balance is observed for soluble PM from Naples samples, since the μmoles of cations turn out to be balanced to the μmoles of anions detected through our analytical procedure, for each washing solution (Figure 2a). On the other hand, the evaluation of the ionic balance of washing solutions related to the species collected in Terni underlines a different behaviour (Figure 1b). Specifically, cations μmoles appear to be higher than the corresponding anions μmoles for five of the twelve sampled tree species (namely, *Robinia pseudoacacia* L. (*R. p.*), *Tilia cordata* Mill. (*T. c.*), *Populus nigra* L. (*P. n.*), *Cedrus atlantica* ((Endl.) Manetti ex Carrière) (*C. a.*) and *Populus tremula* L. (*P. t.*)). This could indicate the presence of anionic species that cannot be detected through our analytical procedure, as it is further suggested by the high WSOC concentrations observed in the washing solutions characterized by the unbalance of ionic species. For instance, WSOC concentration as high as up to 0.59 ± 0.21 mg L^−1^ is measured for *Tilia cordata* L., while ionic balanced species such as *Platanus acerifolia* ((Aiton) Willd.) and *Magnolia grandifolia* L. present in their washing solutions WSOC concentrations as low as 0.010 ± 0.002 mg L^−1^ and 0.0100 ± 0.0005 mg L^−1^, respectively. Since WSOC analysis does not provide any kind of information about the ionic charge of organic species, it makes impossible to use these concentrations in the evaluation of the ionic balance. However, the high concentrations of WSOC detected for *P. tremula, P. nigra, C. atlantica, T. cordata* and *R. pseudoacacia* could be related to the presence of biological matrices on the leaves. Indeed, when studied by SEM/EDX microanalysis, the leaf surfaces of *P. nigra, T. cordata* and *R. pseudoacacia* resulted to be characterized by high accumulation of organic matrices, such as honeydew produced as a response to parasitic infections. For these species, the presence of these organic matrices would be able to totally cover morphological leaves traits, making it impossible to characterize them [18]. 

For *C. atlantica*, the only coniferous species analysed, we hypothesize that the high concentrations of WSOC and the ionic unbalance of its washing solutions may be related to the presence of resins. All these biological matrices may be partially taken in solution, thus affecting the composition of leaves washing solutions.

**Figure 3:**
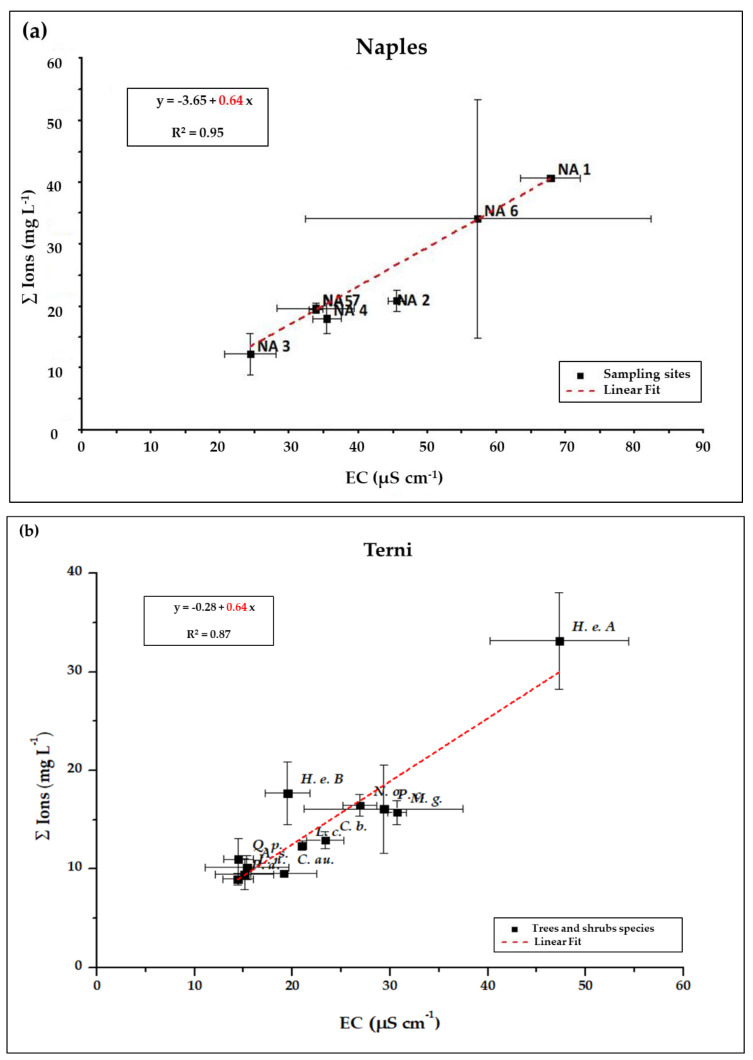
Relationship between ∑ Ions (mg L^−1^) and electrical conductivity (EC)) (µS cm^−1^) of washing solutions from: (**a**) *Q. ilex* (L.) leaves sampled in seven sites within an urban forest in Naples; (**b**) leaves from selected tree and shrub species from an urban park in Terni. Values of ∑ Ions (mg L^−1^) and EC (µS cm^−1^) are obtained as averaged over two replicate branches. Standard deviations are shown. The linear regressions are obtained by considering only the samples characterized by ionic balanced washing solutions.

The total ionic concentrations of washing solutions, ∑ Ions (in mg L^−1^) obtained by ICP-MS, IC and UV-Vis spectrophotometer, are then compared with the corresponding EC (Figure 3). In Naples samples (Figure 3a), except for site NA 6, replicate branches showed a good repeatability, with low standard deviations, for both the EC and the ∑ Ions (standard deviations < 30%). A good linear correlation between the two parameters is obtained (R^2^ = 0.95), with a conversion coefficient *K* = 0.64. In Terni samples (Figure 3b), a good repeatability between the two replicate branches is also obtained, both for EC and ∑ Ions results (with standard deviations between the two replicates < 30%). A linear correlation is still observed between ∑ Ions and EC results over the whole Terni dataset, but with a conversion coefficient much lower with respect to that obtained by the Naples dataset (*K* = 0.44; R^2^ = 0.96; data not shown). However, by considering only the ionic balanced tree and shrubs species (i.e., *P. acerifolia, C. australis, C. bignoides, A. saccharinum, Q. pubescens, M. grandifolia, P. cerasifera, L. nobilis, L. cerasus, N. oleander* and *H. helix*), the conversion coefficient obtained is again *K* = 0.64 (as shown in Figure 3b) with a good linear correlation (R^2^ = 0.87).

This discrepancy could be likely due, again, to the presence of biological organic matrices on the leaves of some of the sampled species in Terni that, partially taken in solution, are able to affect not only their ionic balance but also EC measurements (Figure 2b). Indeed, a conversion coefficient of 0.44 has no counterpart in the literature, while 0.64 is already known as the conversion coefficient to be applied to EC values of aqueous solutions characterized by a prevalence of monovalent ions to obtain the corresponding TDS concentrations [43,53,54]. Our data analytically validate the literature results and confirm the efficiency of this conversion coefficient for most of the species taken in account in this study, both holm oak from Naples and the eleven ionically balanced species from Terni. This suggests that EC measurements could be considered a fast and easy to apply way to quantify the soluble fraction of leaf deposited PM in washing solutions and this deserves particular interest when PM load quantification is performed gravimetrically through V/F, since this procedure cannot provide information on the soluble fraction otherwise. However, the estimation of the soluble PM fraction by EC measurements seems to be reliable only for those species which are not characterized by substantial presence of biological matrices, such as honeydew or resins, on their leaf surfaces. For instance, we have shown that this technique cannot be used with *P. tremula, P. nigra, C. atlantica, T. cordata* and *R. pseudoacacia.*

### 3.2. PM Insoluble Fraction: Chemical Characterization of Membrane Filters and Comparison with V/F Gravimetric Results

**Figure 4:**
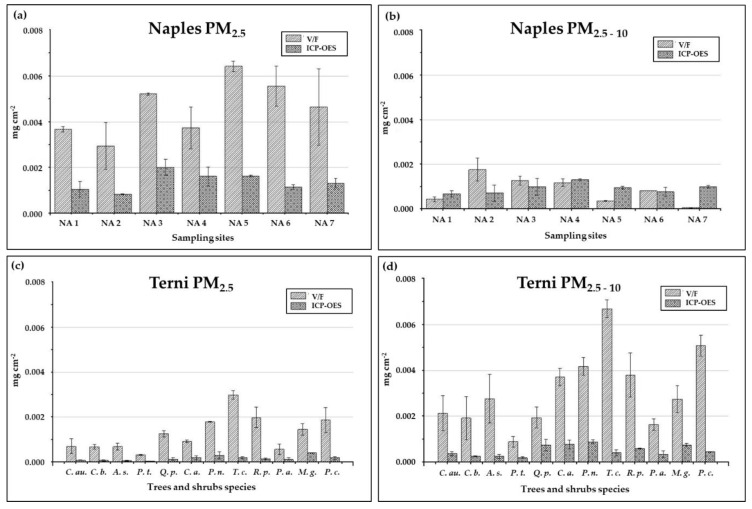
Load of insoluble fraction of leaf deposited particulate matter (PM) (mg cm^−2^) detected gravimetrically (vacuum filtration, V/F) and through the chemical characterization (inductively coupled plasma - optical emission spectrometry, ICP-OES) of PM_2.5_ (**a**,**c**) and PM_2.5-10_ (**b**,**d**) membrane filters used to filter the washing solutions of the collected urban leaves. Int the different panels are reported data for PM2,5 (**a**) and PM_2.5-10_ (**b**) from holm oak leaves collected in Naples and for PM2,5 (**c**) and PM_2.5-10_ (**d**) from different tree species collected in Terni. Tree species are: *Acer saccharinum* (*A. c.*), *Catalpa bignonioides* (*C. p.*), *Cedrus atlantica* (*C. a*.), *Celtis australis* (*C. au.*), *Magnolia grandiflora* (*M. g.*), *Platanus acerifolia* (*P. a.*), *Populus nigra* (*P. n.*), *Populus tremula* (*P. t.*), *Prunus cerasifera* (*P. c.*), *Quercus pubescens* (*Q. p*), *Robinia pseudoacacia* (*R. p.*) and *Tilia cordata* (*T. c.*)). Each bar represents the mean of N = 2 replicates ± standard deviations.

PM insoluble fraction concentrations per unit of leaf area (in mg cm^−2^) obtained through the chemical characterization (ICP-OES) of the membrane filters are shown in Figure 3, compared with the gravimetric results obtained by the same filters. The quantification obtained by the chemical fractionation procedure of the insoluble fraction of PM is generally lower than that obtained gravimetrically, except for the case of the coarse PM particles deposited on the holm oak leaves collected in Naples (Figure 4b). This underestimation of the insoluble PM mass when detected through ICP-OES could be due to three main reasons: (a) the lack of information on the chemical speciation of PM elemental components [56,57,58]; (b) the presence of insoluble organic species that cannot be detected by ICP-OES, such as black carbon particles and polycyclic aromatic hydrocarbons (PAHs) emitted by anthropogenic emission sources like those deriving from combustive processes (e.g., vehicular traffic, domestic heating, biomass, combustion, industrial plants and incinerators) [59,60]; (c) the presence of organic insoluble matrixes of biological origin on the leaf surfaces.

For Naples case study, where leaves were collected during the winter season, we could suggest that fine PM (PM_0.2–2.5_) is partially composed by insoluble organic species deriving from domestic heating. These pollutants are known to be mainly associated to airborne particles in the fine size class (especially ≤1 μm) [59,60] and, thus, it would have no (or very little) impact on the coarse particle fraction load, as observed.

A similar effect is not expected (and, indeed, not observed) for Terni samples, since the sampling has been conducted during summer. However, the sampling site has been selected as strongly exposed to the air pollution coming from a steel factory, and an incinerator is also present close to the city. The high discrepancy observed in Terni dataset, at every size fraction and for each studied species, between the quantification obtained by the two techniques rather suggest that ICP-OES is intrinsically unable to quantitatively measure the insoluble PM characterizing Terni air pollution, likely due to a superimposition of the first two of the above mentioned reasons. A minor contribution of biological matrixes to the gravimetric values can be also inferred, since four over the five species having gravimetric PM_2.5–10_ load higher than 0.003 mg cm^−2^ are those previously described as showing no ionic balance in their washing solutions (*C. atlantica*, *P. nigra*, *T. cordata* and *R. pseudoacacia*).

### 3.3. Reliability of Chemical Fractionation Procedure for the Evaluation of PM Emission Sources

Principal Component Analyses (PCAs) based on correlations were performed by using the elemental concentrations per unit leaf area (mg cm^−2^) as obtained by the chemical fractionation procedure. In order to prove the efficiency of the chemical fractionation procedure in increasing the selectivity of PM elemental components as specific source tracers, for each element, we considered only the concentrations of the solubility fraction (either water-soluble or insoluble one) in which it has its relative highest concentration. Indeed, these fractions correspond to those in which the elemental components are more likely to be emitted by PM sources. Moreover, the elemental concentrations in the selected fractions are also those showing the highest variability between the different samples, per each element. For the insoluble fraction, the concentrations obtained by merging both size fractions (PM_2.5_ and PM_2.5–10_) are considered, thus corresponding to total PM_10_.

**Figure 5:**
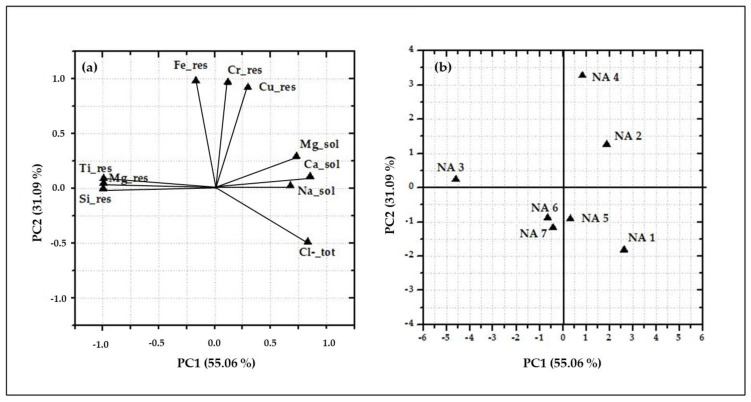
Biplots of the factor coordinates (**a**) and of factor scores (**b**) of the first two principal components (PCs) obtained by correlation-based principal component analysis (PCA) of the concentrations per unit leaf area in the water-soluble fraction of Ca, Mg, Na, Cl and in the insoluble fraction of Cr, Cu, Fe, Mg, Si and Ti, detected at the seven sampling sites within the urban forest in Naples.

The factor coordinates and scores obtained by the PCA for Naples data are reported in Figure 5, where the first two principal components (PCs) are shown. As input variables, the concentration per unit leaf area of the water-soluble fraction of Ca, Mg, Na and Cl together with the ones of the insoluble fraction of Cr, Cu, Fe, Si and Ti are used. The eigenvalues of the two first PCs are at 55.06% and 31.09%, respectively, representing the 86.15% of the total variance. PC1 discriminates sampling sites characterized by high concentration of Mg, Si and Ti in their insoluble fraction (namely, NA 3 and, to a minor extent, NA 6 and NA 7), from those characterized by the presence of Ca, Mg, Na and Cl in the PM soluble fraction (NA 1 and NA 5). PC2 discriminates the sites characterized by high concentrations of Cr, Cu and Fe in the insoluble fraction (NA 2 and NA 4). The clustering of NA 1 and NA 5, discriminated by water-soluble concentrations of Ca, Cl, Mg and Na, which are known elemental components of marine salts, confirms the impact of the South-West marine breeze at these sites. On the other hand, the clustering of NA 2 and NA 4 underlines the emission impact of vehicular traffic at the sites located at high proximity to a trafficked street. Indeed, Cr, Cu and Fe are known to be related to vehicular traffic mechanical-abrasive emission processes, being component of brakes, tire dust and vehicle components [49,61,62]. Leaves collected from NA 2 seemed also to be affected by the South-West marine breeze with relative higher concentrations of marine salts components. Finally, the relative high concentration of Mg, Si and Ti in the insoluble fraction shown by NA 3, together with NA 6 and NA 7, could underline the role of soil resuspension, being these elements often associated to soil compounds. Therefore, the utilization of holm oak leaves, sampled from seven different sampling sites, with different exposure, resulted effective in the identification of the main emission sources acting in this area. In fact, leaves collected from each site, were characterized by distinctive concentrations of those elements, which are known to be tracers for specific emission sources (both natural and anthropogenic). These results prove the efficiency of the chemical fractionation procedure for increasing the selectivity of inorganic PM elemental components as specific source tracers and the utilization of holm oak leaves as efficient passive filters for PM monitoring. Most interestingly, the site clustering obtained by the chemical fractionation data recalls the one previously obtained for the same sampling sites by analysing the holm oak leaves by SEM/EDX data [31].

**Figure 6:**
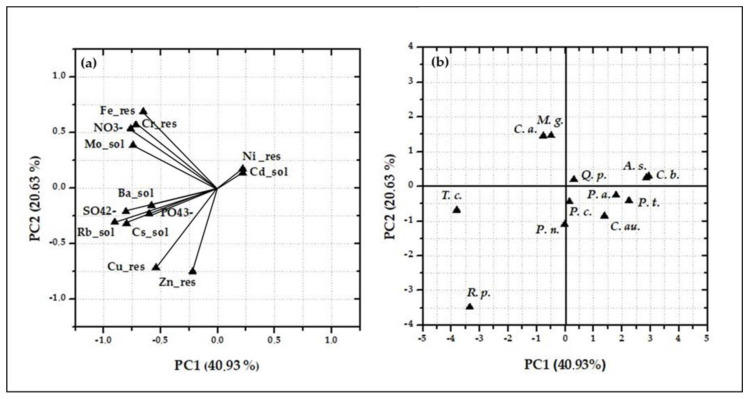
Biplots of the factor coordinates (**a**) and of factor scores (**b**) of the first principal components (PCs) obtained by correlation-based principal component analysis (PCA) of the concentrations per unit leaf area in the water-soluble fraction of Ba, Cd, Cs, Mo, Rb, NO_3_^2−^, SO_4_^2−^, PO_4_^3−^ and in the insoluble fraction of Cr, Cu, Fe, Ni and Zn, as obtained by the twelve tree species sampled in the urban park in Terni. Tree species are: *Acer saccharinum* (*A. c.*), *Catalpa bignonioides* (*C. p.*), *Cedrus atlantica* (*C. a*.), *Celtis australis* (*C. au.*), *Magnolia grandiflora* (*M. g.*), *Platanus acerifolia* (*P. a.*), *Populus nigra* (*P. n.*), *Populus tremula* (*P. t.*), *Prunus cerasifera* (*P. c.*), *Quercus pubescens* (*Q. p*), *Robinia pseudoacacia* (*R. p.*) and *Tilia cordata* (*T. c.*).

The factor coordinates and scores obtained by the PCA for Terni tree data are reported in Figure 6, where the first two PCs are shown. As input variables, the concentrations per unit leaf area of the water-soluble fraction of Ba, Cd, Cs, Mo, Rb, NO_3_^2−^, SO_4_^2−^, PO_4_^3−^ and of the insoluble fraction of Cr, Cu, Fe, Ni and Zn, are used. The eigenvalues of the first two PCs are at 40.93% and 20.63%, respectively, representing the 61.56% of the total variance. Based on the factor coordinates (Figure 6a), clear correlations exist among elemental concentrations per unit leaf area, such as: water-soluble Cd and insoluble Ni (positive PC1 values); water-soluble Ba, Cs, Rb, SO_4_^2−^ and PO_4_^3−^ (negative PC1 values); water-soluble Mo, insoluble Fe and Cr together with NO_3_^2−^ (positive PC2 values); insoluble Cu and Zn (negative PC2 values). These elemental clusters can be considered as representative of the different PM emission sources present in Terni: water-soluble Mo and insoluble concentrations of Cr, Fe and Ni, were associated to the emission impact of the steel plant [46,49], since these elements are used as chemical components in the stainless-steel production to increase its ductility, strength and toughness [63]; water-soluble Cs and Rb and ionic species such as SO_4_^2−^ and PO_4_^3−^ could be related to the influence of biomass burning [47,64]; and finally, insoluble Cu and Zn are known to be elemental tracers related to the impact of mechanical and abrasive processes connected to vehicular traffic, being component of brakes and other vehicle parts [65,66,67]. However, the identification of the main emission sources is only slightly reflected in the case discrimination (Figure 6b): most of the tree species (eight over twelve) are clustered together in the positive PC1 region, without differentiation in PC2. 

This is not surprising, since trees were sampled in the same park and with the same orientation with respect to the main PM source (i.e., the Terni steel factory). However, four species show peculiar PM elemental compositions and are, thus, discriminated: *T. cordata* (negative PC1), *C. atlantica* and *M. grandifolia* (positive PC2), and *R. pseudoacacia* (negative PC2). Interestingly, three of them (*T. cordata*, *C. atlantica* and *R. pseudoacacia*) are among those characterized by ionically unbalanced washing solutions, likely due to the accumulation of biological and organic matrices on their leaf surfaces. T. cordata leaves are characterized by the highest concentrations of elemental source tracers known to be connected to the emission of biomass burning (i.e., water-soluble Cs and Rb and ionic species such as SO_4_^2−^ and PO_4_^3−^). As described previously, this specific emission sources are known to be associated mainly to airborne particles in the fine fraction [59,60]. High accumulation of fine particles on the leaves of *T. cordata* has been previously reported by SEM/EDX microanalysis results [18] and this could be due to the increased PM retention capacity of its leaves because of the presence of honeydew. The leaves of *C. atlantica* and *M. grandiflora* show a relatively high affinity for the emission of the steel plant metals (i.e., insoluble Fe and Cr together with NO_3_^2−^ and water-soluble Mo). Both were reported to have high affinity with fine PM [18], likely due to the presence of resins (*C. atlantica*) and trichomes (*M. grandiflora*) on their leaves. *R. pseudoacacia* leaves were mainly characterized by high concentrations of elements associated to the emission of abrasive and mechanical traffic-related emissions, known to be associated to particles in the coarse fraction [65,66,67]. In Sgrigna et al. *R. pseudoacacia* leaves resulted to be characterized by rough surfaces with deep grooves larger than 1 μm that may be connected to the higher retention of coarse particles [18]. Thus, the PCA performed on Terni tree data proved again the chemical fractionation efficiency for the individuation of the elemental source tracers of the main anthropogenic PM emission sources acting in the area and also indicated that, despite the twelve tree species leaves were sampled in the same location, some of them show selective affinity for specific PM elemental component. This may be due to the different interaction of particles with peculiar chemical and physical characteristics and the tree leaf species-specific traits, which is an interesting aspect which still deserve further investigations [18].

### 3.4. Total PM Loads and Single Element Concentrations Per Unit Leaf Area: Comparison between Chemical Fractionation and Scanning Electron Microanalysis

**Figure 7:**
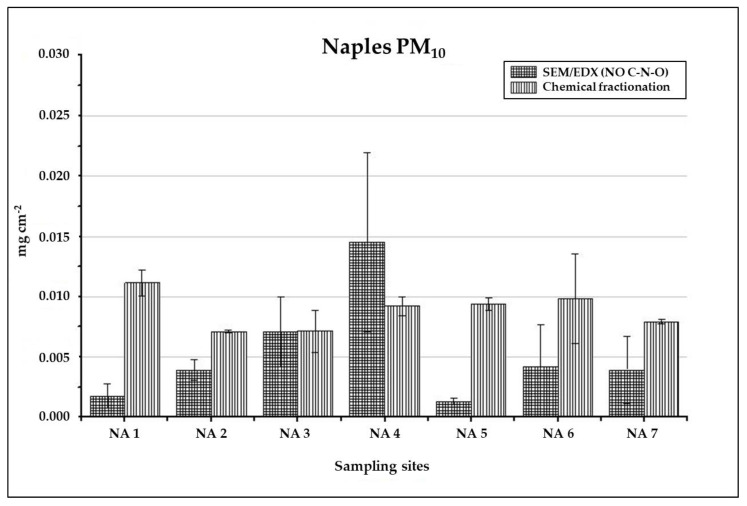
Comparison between total PM load (mg cm^−2^) detected through the chemical fractionation procedure (Soluble + Insoluble fraction) and through SEM/EDX, in Naples samples. Each bar represents the mean of N = 2 replicates ± standard deviations.

The total inorganic PM_10_ loads of the holm oak leaves collected at the Naples sampling sites have been estimated by summing together the total soluble and insoluble fraction loads obtained by the chemical fractionation procedure, and they are shown in Figure 7. 

WSOC has been excluded by the total PM_10_ load estimation, since it is impossible to discriminate biological soluble organic species produced by the plant from organic species related to leaf deposited PM. The PM_10_ loads obtained by analysing leaves collected at the same sampling sites by SEM/EDX are also reported in Figure 7, for comparison. For the same reason as above, the relative percentages of C, N and O have been excluded by the calculation of PM_10_ loads also in this case [31]. Most interestingly, the PM_10_ loads obtained by the two techniques are in the same order of magnitude at every site, thus confirming the reliability of our fractionation procedure in estimating PM load. Differences in the total PM_10_ load determination among the two techniques are obtained at sites NA 1 and NA 5, where the SEM/EDX PM loads are significantly lower not only in comparison to the corresponding chemical fractionation ones but also to all the SEM/EDX loads obtained at the other sites. As shown previously, sites NA 1 and NA 5 are windward with respect to the marine breeze and are characterized by high levels of Na and Cl and low Fe concentration, this could lead to an underestimation of PM load by SEM/EDX, as previously discussed [31]. Moreover, a general underestimation of fine particles (PM_2.5_) is expected from SEM/EDX, due to the limitation of imaging resolution.

**Figure 8:**
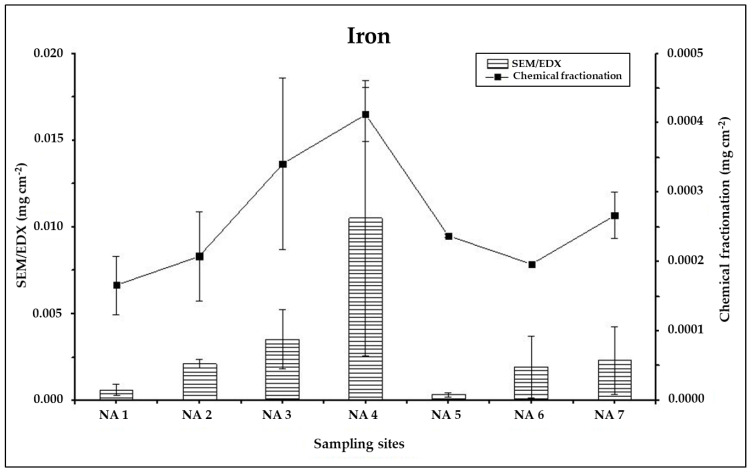
Comparison between total PM load (mg cm^−2^) detected through the chemical fractionation procedure (Soluble + Insoluble fraction) and through SEM/EDX, in Naples samples. Each bar represents the mean of N = 2 replicates ± standard deviations.

This is further suggested by the comparison of concentrations of single elemental components: while significant correlations are obtained between the elemental concentration of Fe (ρ = 0.91) and Si (ρ = 0.78) in the insoluble fraction of coarse PM (PM_2.5–10_) and in the SEM/EDX data, no significant correlations (Pearson’s ρ < 0.75) are found when SEM/EDX results are compared with the elemental concentrations of Al, Cr, Cu, Fe, Si and Ti in the insoluble fine fraction (PM_2.5_). However, as previously said, the PM_2.5–10_ size class is the one which mainly drive the total PM_10_ amount. Indeed, a significant correlation (ρ = 0.87) is still obtained when comparing the PM_10_ Fe concentration per unit leaf area obtained by the chemical fractionation with that from SEM/EDX (Figure 8): despite the concentrations detected through the chemical fractionation are an order of magnitude lower than the ones by SEM/EDX, an analogous behaviour of the Fe concentration as a function of the sampling site is obtained. The only exception is NA 5 site, for which an underestimation of the Fe content by SEM/EDX can be thus suggested.

## 4. Conclusions

This study focused on the potential of chemical fractionation procedure in the characterization of inorganic leaf deposited PM, when investigating both its water-soluble and insoluble fraction, as obtained by leaf V/F procedure. The study was composed by two sub-studies mainly aimed at evaluate the sensitivity of the proposed procedure to different PM sources (Naples data from holm oak leaves sampled at seven different sites) and the suitability of different plant species to be used as passive filters for PM monitoring (Terni data from twelve tree and four shrub species, from the same site).

From the Naples campaign, we have shown that: the PM water-soluble fraction quantification obtained by the chemical fractionation procedure is proportional to the washing solution EC (*K* = 0.64); the quantification of the insoluble fraction of PM (by ICP-OES) is consistent with the gravimetric results for the coarse particle fractions; by summing the two fractions, PM_10_ loads consistent with those previously determined by SEM/EDX for the same sites can be obtained. Moreover, by using single elemental concentrations from both the water-soluble and insoluble fraction as input variable of a multivariate analysis, the efficiency of this analytical approach to increase the selectivity of elements as specific source tracers has been proved.

From the Terni campaign, we have obtained that: for eleven over the sixteen tested species, the PM water-soluble fraction quantification obtained by the chemical fractionation procedure is still proportional to the washing solution EC, with the same conversion coefficient as above; the chemical characterization of the insoluble fraction of PM is not reliable, since it appears to be highly dependent on the amounts of organic compounds in the PM itself. Nevertheless, the single elemental concentrations as obtained by the chemical fractionation of both the water-soluble and insoluble fraction of leaf deposited PM appear to be efficient source tracers also in the case of Terni. Interestingly, some species emerge as not suitable to be used as passive filters for PM monitoring (i.e., *P. tremula*, *P. nigra*, *C. atlantica*, *T. cordata*, *R. pseudoacacia* and, to a minor extent, *M. grandiflora*), likely due to the presence of biological and organic matrices (such as honeydew and resins), as well as peculiar surface traits, on their leaves. However, these characteristics seems to increase, at the same time, their PM capturing capability.

Thus, the total of our results confirms the potential of leaves as a low-cost alternative for monitoring PM pollution, suggests that EC of leaf washing solutions is a fast and easy to apply technique to estimate the water-soluble PM content, and that the proposed chemical fractionation procedure is a reliable, analytical tool to study leaf deposited PM in connection with potential sources. The main limitations of the proposed approach reside in its inability in detecting organic compounds (which may be important in the insoluble PM fraction) and in its dependency on the tree species; both deserving the need of further investigations. In particular, the interactions between airborne particles with specific chemical-physical characters and leaves with species-specific surface properties could deserve interest to develop future air pollutant-specific nature-based solutions, in which the planted tree species will be choose as a function of the affinity they have with the most abundant air pollutants and emission sources acting in the area.
